# Evaluation of Anti-HIF and Anti-Angiogenic Properties of Honokiol for the Treatment of Ocular Neovascular Diseases

**DOI:** 10.1371/journal.pone.0113717

**Published:** 2014-11-25

**Authors:** Divya Teja Vavilala, V. K. Chaithanya Ponnaluri, Debolina Kanjilal, Mridul Mukherji

**Affiliations:** Division of Pharmaceutical Sciences, School of Pharmacy, University of Missouri-Kansas City, Kansas City, Missouri, United States of America; Children's Hospital Boston & Harvard Medical School, United States of America

## Abstract

Pathological activation of the hypoxia-inducible-factor (HIF) pathway leading to expression of pro-angiogenic genes, such as vascular endothelial growth factor (VEGF), is the fundamental cause of neovascularization in ocular ischemic diseases and cancers. We have shown that pure honokiol inhibits the HIF pathway and hypoxia-mediated expression of pro-angiogenic genes in a number of cancer and retinal pigment epithelial (RPE) cell lines. The crude extracts, containing honokiol, from *Magnolia* plants have been used for thousands of years in the traditional oriental medicine for a number of health benefits. We have recently demonstrated that daily intraperitoneal injection of honokiol starting at postnatal day (P) 12 in an oxygen induced retinopathy mouse model significantly reduced retinal neovascularization at P17. Here, we evaluate the mechanism of HIF inhibition by honokiol in RPE cells. Using chromatin immunoprecipitation experiments, we demonstrate that honokiol inhibits binding of HIF to hypoxia-response elements present on VEGF promoter. We further show using a number of *in vitro* angiogenesis assays that, in addition to anti-HIF effect, honokiol manifests potent anti-angiogenic effect on human retinal micro vascular endothelial cells. Our results suggest that honokiol possesses potent anti-HIF and anti-angiogenic properties. These properties of honokiol make it an ideal therapeutic agent for the treatment of ocular neovascular diseases and solid tumors.

## Introduction

Pathological neovascularization (NV), where abnormal vasculature originate from existing blood vessels, is observed in most solid tumors and in retinal ischemic diseases, such as diabetic retinopathy (DR), retinopathy of prematurity (ROP), retinal vein occlusion (RVO), etc. [1], [2], [3]. Despite different primary cause and clinical manifestations, these diseases share a common molecular feature, *i.e.* hypoxia mediated activation of the hypoxia inducible factor (HIF) pathway. Central to this pathway is the heterodimeric HIF transcription factor. This heterodimeric transcription factor consists of HIF-α (HIF-α has three subunits: HIF-1α, HIF-2α, and HIF-3α) and HIF-β, of which HIF-α is the oxygen sensitive counterpart [4], [5], [6]. The oxygen sensitivity of HIF-α under normoxic conditions is regulated by hydroxylation of proline and asparagine residues (HIF-1α-Pro402, -Pro564 and -Asn803) by oxygen dependent dioxygenases/hydroxylases: prolyl hydroxylase (PHD1-3) [7], [8] and factor-inhibiting-HIF (FIH) hydroxylase [9], respectively. Proline hydroxylation allows Von Hippel-Lindau (pVHL) tumor suppressor to recruit HIF-α for proteosomal degradation in the cytoplasm [10]. If any HIF-α escapes cytoplasmic degradation and translocates to the nucleus, then asparagine hydroxylation prevents its binding to co-activator (p300/CBP), thereby hindering HIF mediated transcription [11]. However, under hypoxic conditions HIF hydroxylases become inactive, ceasing HIF-α hydroxylation. This allows translocation of HIF-α in to the nucleus and its dimerization with HIF-β, forming the active HIF complex. Activated HIF binds to hypoxia response element (HRE) present in the promoters of hypoxia response genes including many critical proteins involved in angiogenesis [12].

Important HIF regulated pro-angiogenic factors include vascular endothelial growth factor (VEGF), insulin growth factor (IGF-1), fibroblast growth factor (FGF), stromal derived growth factor-1 (SDF-1), placental growth factor (PlGF), platelet-derived growth factor (PDGF), etc [13], [14]. These factors act in an orchestrated fashion to initiate pathological retinal NV. Consistently, in addition to surgeries, a number of anti-angiogenic therapies targeting VEGF (*e.g.* Macugen, Lucentis, and VEGF Trap) have been approved [15]. However, all current therapies have significant limitations and side-effects [16], [17]; *e.g.* anti-VEGF therapies require repeated administration at high cost, and yet, only offer temporary respite from vascular leakage. Thus, there remains a critical need for the development of an effective therapeutic agent, which can complement current treatments for ocular NV. Inhibition of the HIF pathway, the master regulator of ocular NV, offers an attractive therapeutic strategy.

We screened a number of known phytochemicals from traditional medicine for their effect on the HIF pathway and identified honokiol as a candidate molecule [18]. Honokiol is a biphenolic phytochemical extracted from the bark and/or seed cones of *Magnolia* plants. The crude extracts, containing honokiol, have been extensively used in the traditional oriental medicine [19]. We have shown that pure honokiol inhibits the HIF pathway and hypoxia-mediated expression of pro-angiogenic genes under hypoxic conditions in a number of cancer and retinal pigment epithelial (RPE) cell lines [18]. In addition, honokiol is a neuroprotective agent with no known toxicity [20], [21] and has a better efficacy profile (anti-HIF IC_50_/anti-proliferation IC_50_) [22], [23] than other HIF inhibitors that are currently evaluated for ocular diseases [24], [25]. We also demonstrated that daily intraperitoneal injection of honokiol starting at postnatal day (P) 12 in an Oxygen Induced Retinopathy (OIR, which mimics ROP in humans) mouse model significantly reduced retinal neovascularization at P17 [23]. Importantly, administration of honokiol also prevented the oxygen-induced central retinal vaso-obliteration and promoted normal revascularization of the retinal vascular plexuses. Here, we evaluate the mechanism of HIF inhibition by honokiol in RPE cells. We further show using a number of *in vitro* angiogenesis assays that, in addition to anti-HIF effect, honokiol manifests potent anti-angiogenic effect on human retinal micro vascular endothelial cells (hRMVECs).

## Materials and Methods

### Cell culture and exposure of cells to hypoxia

Human RPE cell lines, ARPE-19 and D407 (a generous gift from Dr. Richard Hunt, University of North Carolina, Chapel Hill), and cervical cancer cell line, HeLa, were cultured as previously reported [26], [27]. Human Retinal Microvascular Endothelial Cells (hRMVECs) were purchased from Angio-Proteomie (Boston, MA) and were cultured in ENDO-growth media (Angio-Proteomie). Honokiol was dissolved in DMSO and was added to the cells. In control samples 0.1% of DMSO, corresponding to the DMSO concentration in the cells treated with highest honokiol concentration, was added. Cells were exposed to hypoxic condition (*i.e.* 1% O_2_, 5% CO_2_, and 94% N_2_) in a bactron anaerobic chamber as previously reported [26], [27]. Since under physiological conditions posterior segment of the eye experiences an oxygen tension between 8–22 mm Hg (*i.e.* 1–4% O_2_) [28], [29], we performed all our cell-based hypoxia experiments at 1% oxygen. For the qPCR experiments, total RNA was isolated from RPE cells grown under normoxia, hypoxia, or hypoxia treated with honokiol and quantified as described in our previous publications [26], [27].

### Anti-HIF IC_50_ value of honokiol

A HIF reporter assay was performed in biological duplicates and experimental triplicates to determine the anti-HIF IC_50_ value of honokiol. To perform this experiment, HeLa cells were cultured in a 96-well plate at a density of 8×10^3^ cells/well and incubated overnight. Cells were co-transfected with 5HRE-ODD-luc plasmid (a generous gift from Dr. Hiroshi Harada, Kyoto University, Japan) and pSVRenilla using Lipofectamine 2000 (Invitrogen, Grand Island, NY) for 6 h. Later the transfection mix was replaced with regular media and the cells were incubated for 24 h. The transfection efficiency was optimized to ≈80%. Cells were further treated with 1 mM CoCl_2_ (hypoxia-mimetic agent) under normoxia and different honokiol concentrations (0.2–100 µM), and were incubated for 12 h. Following the incubation cells were processed using Dual-Luciferase Reporter Assay System (Promega, Madison, WI) according to manufacturer's instructions. For each well, firefly and renilla luminescence were measured using Analyst GT micro titer plate reader (Molecular Devices, Sunnyvale, CA). The firefly luminescence obtained from each well was normalized with its corresponding renilla luminescence values to obtain the firefly luminescence/renilla luminescence value. To evaluate the anti-HIF effect of honokiol, firefly luminescence/renilla luminescence values of honokiol was normalized with the values of 1 mM CoCl_2_ treated wells. The obtained relative luciferase activity values were plugged into Graph Pad Prism software to obtain anti-HIF IC_50_ value of honokiol.

### Immunoblot analysis

To perform immunoblot analysis of HIF-1α; D407 and ARPE-19 cells were exposed to normoxia, hypoxia and hypoxia with 20 µM honokiol for 12 h. Immunoblot for each condition was performed in biological duplicates and experimental triplicates. Following the exposure, cells were washed with 1× PBS and harvested using ice-cold RIPA buffer containing protease inhibitor cocktail. Due to the short half-life (5–10 min) of HIF-1α protein, cells exposed to hypoxic conditions were processed inside the hypoxia chamber. Cell lysates were collected and the protein concentrations were determined using Bradford assay kit (Bio-Rad, Hercules, CA). Twenty µg of cell lysate was resolved on a 10% SDS-PAGE gel and then transferred onto a PVDF nitrocellulose membrane (Bio Rad apparatus was used for the semidry transfer). The membrane was blocked with 5% BSA, washed with 1× TBS containing 0.1% tween 20 (TBST) and immunoblotting was performed using anti-HIF-1α polyclonal antibody (dilution of 1∶1000) (ab2185, abcam, Cambridge, MA) and anti-HIF-1α monoclonal antibody (dilution of 1∶400) (ab1, abcam). After multiple TBST washes, peroxidase-conjugated IgG secondary antibody (dilution of 1∶1000) was added. Further, the blot was washed with TBST three times and developed by adding BM Chemiluminiscence reagent (Roche, Indianapolis, IN). The images were captured using Chemi-Doc XRS imager equipped with Quantity One software (Bio-Rad). The membrane was stripped using stripping buffer and re-probed for β-actin using rabbit anti β-actin antibody (dilution of 1∶2000) (Aviva Systems Biology, San Diego, CA) and peroxidase-conjugated goat anti-rabbit IgG (1∶1000) secondary antibody. Densitometry analysis was performed on all (n = 6) the images using Quantity One software. The intensities of HIF-1α were normalized to their corresponding β-actin intensities and represented as a bar graph.

### Immunofluoroscence analysis

Immunofluoroscence studies were performed to evaluate the effect of honokiol on translocation of HIF-1α from cytoplasm to the nucleus and on HIF-1α and HIF-β co-localization in the nucleus. D407 cells were seeded in a 12-well plate on cover slips, at a density of 1×10^5^ cells/well and incubated overnight before exposing to normoxia, hypoxia and hypoxia with 20 µM honokiol for 5, 7, 9, and 12 h. After exposing cells to different conditions they were washed with 1× PBS and fixed with 4% paraformaldehyde and 0.5% triton X-100 for 10 min. After incubation, the solution was aspirated and washed with 1× PBS. The cover slips were then transferred to a fresh 12-well plate and blocked with 5% goat serum (Jackson Laboratory, Bar Harbor, ME) in 1× PBS containing 0.1% triton X-100 (PBST). Cells were thoroughly washed with 1× PBS and then incubated overnight with mouse anti-HIF-1α antibody (dilution of 1∶200) (ab1, abcam) at 4°C. Following this, cells were washed thoroughly with PBST and then incubated with anti-mouse alexa flour 488 antibody (dilution 1∶200) (Jackson Laboratory) for 2 h at 37°C. DAPI (USB, Cleveland, OH) at a concentration of 0.01 µg/mL was added for the last 45 min in the above incubation. Cells were washed with PBST and the coverslips were mounted on glass slides using fluoroshield mounting media (Sigma, St. Louis, MO). Confocal images for each slide were taken from 5 different positions at 100× magnification using Leica TCS SP5 II (Leica Microsystems, Buffalo Grove, IL). Green channel represents HIF-1α and blue channel represents DAPI. Image J with LOCI plugin was used to process confocal images. Further, “just another co-localization program” (JACoP) plugin for ImageJ was used to determine the extent of translocation of HIF-1α in the nucleus. Using the JACoP plugin, Coste's automatic threshold approach was run to evaluate Mander's coefficients M1 and M2. M1 represents the ratio of the sum of the signal intensity from blue pixels (DAPI), where the intensity for green pixels (HIF-1α) was greater than zero, to the total blue pixel intensity (extent of DAPI associated with HIF-1α) and M2 represents vice-versa. Two-tailed Student t-test was performed to evaluate the statistical significance.

To study HIF-1α and HIF-β co-localization in the nucleus the above-discussed procedure was followed with a variation. Cells were incubated overnight at 4°C with mouse anti-HIF-1α antibody (dilution of 1∶100) (ab1, abcam) and rabbit anti-HIF-1β antibody (dilution of 1∶500) (ab2, abcam). Following the washes they were incubated with anti-mouse alexa flour 488 antibody (dilution 1∶100) (Jackson Laboratory), anti-rabbit alexa flour 585 antibody (dilution 1∶500) (Jackson Laboratory) and DAPI (USB). Confocal images were taken where blue channel corresponds to DAPI, green channel corresponds to HIF-1α and red channel corresponds to HIF-β. LOCI plugin and co-localization program (JACoP) plugin for ImageJ were used to evaluate the extent of co-localization of HIF-1α and HIF-β. Coste's automatic threshold approach program was run to evaluate Mander's coefficients M1 and M2. M1 represents the ratio of the sum of the signal intensity from red pixels (HIF-β), where the intensity for green pixels (HIF-1α) is greater than zero, to the total red pixel intensity (extent of HIF-β associated with HIF-1α) and M2 represents vice-versa. Two-tailed Student t-test was performed to check for statistical significance.

### Chromatin immunoprecipitation (ChIP)-quantitative PCR (qPCR)

To perform ChIP, SimpleChIP Chromatin IP Kit Agarose Beads (Cell Signaling Technology, Boston, MA) was used. D407 cells were exposed to different treatment conditions (normoxia, hypoxia and hypoxia with 20 µM honokiol) for 16 h. Cells were processed as per manufacturer's protocol. Briefly, cells were fixed by adding 1% formaldehyde to the media and later quenched by adding glycine. Following this, cells were washed with PBS and lysed in lysis buffer. Micrococal nuclease (7.5 µL) was added to digest the chromatin into 300–900 bp DNA/protein complex. Lysate was then sonicated to break the nuclear membrane and centrifuged to obtain clarified supernatant containing the chromatin material. Chromatin (30 µg) was subjected to immunoprecipitation (IP) by incubating overnight with different antibodies [anti-HIF-1α (ab2185, abcam), IgG (cell signaling technology)] and no antibody at 4°C. Before the addition of antibodies 2% of the chromatin was set aside and used as the input DNA. ChIP-Grade Protein G Agarose Beads were added to precipitate the antibody-protein-DNA complex. Following this, the complex was reverse cross-linked using proteinase K at 65°C to obtain the IP DNA, which was purified using standard procedures. In the IP DNA, relative abundance of DNA sequence from the VEGF promoter region was analyzed by qPCR using LightCycler 480 qPCR instrument (Roche Diagnostics Corporation). The primer sequences used are 5′-AGACTCCACAGTGCATACGTG-3′ (sense) and 5′-AGTGTGTCCCTCTGACAATG-3′ (anti-sense) [30]. qPCR was performed in two biological duplicates (independent ChIP experiments) and three experimental replicates (n = 6). The enriched DNA after qPCR was represented as % input. It was calculated using 100 * 2^−**Δ**Ct^ method where **Δ**C_t_ = [Ct IP]−[Ct 2% Input−5.4]. The number 5.4 is the dilution factor calculated for 2% input.

### Enzyme-linked immunosorbent assay (ELISA)

In order to evaluate the effect of honokiol on VEGF secreted by D407 cells into the media, ELISA was performed using human VEGF ELISA kit (RayBiotech, Inc., Norcross, GA). To perform this assay, D407 cells were exposed to 12, 24, and 36 h under hypoxia with or without 20 µM honokiol. At each time point the spent media was collected and analyzed for the secreted VEGF following the manufacturer's protocol. In brief, this is an indirect sandwich ELISA where the plate is pre coated with anti-VEGF antibody. Standards (known concentrations of VEGF) and spent media were added to each well and incubated for 2.5 h at 25°C. Bound VEGF was sandwiched with biotinylated anti-VEGF antibody and incubated for 1 h at 25°C. To this streptavidin bound horseradish peroxidase was added which converted the chromogenic substance 3, 3′, 5, 5′-Tetramethylbenzidine (TMB) (present in the substrate solution) into a colored product. The absorbance of the colored product was measured at 450 nm using Analyst GT (Molecular Devices), which directly correlates with the amount of VEGF present in the media.

### Anti-proliferative IC_50_ value of honokiol

hRMVECs were plated in a 96-well plate at a density of 1×10^4^ cells/well with varying concentrations (0.4–180 µM) of honokiol. In the wells with no honokiol, 0.5% DMSO was added that corresponds to the amount of DMSO in the highest concentration of the drug. The cells were incubated for 24 h after which, 100 µL fresh media containing 10 µL of premixed WST-1 reagent was added to each well according to the manufacturer's protocol (Clontech, Mountain View, CA). After 30 min incubation, absorbance was measured at 450 nm using Analyst GT (Molecular Devices). The absorbance values were plugged into Graph Pad Prism software to calculate the anti-proliferative IC_50_ value of honokiol on hRMVECs.

### hRMVEC proliferation assay

This assay was performed to study the effect of VEGF (secreted by D407 cells under normoxia, hypoxia, or hypoxia with honokiol) on the proliferation of hRMVECs. hRMVECs were plated in a 96-well plate at a density of 3×10^3^ cells/well and incubated overnight. After the incubation ENDO-growth media was replaced with the incubation media. The incubation media is ENDO-basal media with either varying concentrations of VEGF (2–16 ng/mL) or the spent media collected after exposing D407 cells to different treatment conditions for 36 h (normoxia, hypoxia and hypoxia treated with 20 µM honokiol). Before incubating cells with the spent media, VEGF levels were quantified using ELISA, after which the spent media was diluted 1000 times (for VEGF levels to fall in the range of 2–16 ng/mL). hRMVECs have a doubling time of 2–3 days hence the assay was performed for 3 days. After which the media was removed and the cells were incubated with 10 µL of the premixed-WST-1 cell proliferation kit (Clontech). Absorbance was measured at 450 nm using Analyst GT (Molecular Devices). Error bars are represented as percent standard error. Two-tailed Student t-test was performed.

### hRMVEC migration assay

Oris Cell Migration Assay-Collagen I coated kit (Platypus Technologies, Madison, WI) was used to evaluate the inhibitory effect of honokiol on VEGF-induced migration of hRMVECs. The assay was performed according to the manufacturer's instructions. Briefly, 100 µL of suspended cells (ENDO-growth media) were loaded in each well to achieve 4×10^4^ cells/well. The cells were allowed to adhere for 6 h at 37°C and 5% CO_2_. After cells were attached, stopper from each well was removed except from the wells designated as reference in which the stoppers remained until results were read (t_0_). Next, the cells were washed with ENDO-growth media, followed by treatment for 16 h with different media conditions: ENDO-basal media, ENDO-growth media, ENDO-growth media supplemented with 50 ng/mL VEGF, and ENDO-growth media supplemented with 50 ng/mL VEGF and different concentrations of honokiol (2.5–40 µM). Following the incubation, stoppers from reference wells were removed, washed with 1× PBS, after which they were incubated with calcein AM for 30 min at 37°C. The solution containing calcein AM was aspirated and the cells were suspended in 1× PBS. Images were obtained using a fluorescence microscope and extent of cell migration was evaluated by comparing them to reference wells.

### *In vitro* angiogenesis assay

*In vitro* angiogenesis assay was performed to evaluate the effect of honokiol on the ability of hRMVECs to form tubes on basement membrane, under the trigger of pro-angiogenic factors (VEGF). To carry out this assay, *in vitro* angiogenesis tube formation kit was used (Trevigen, Gaithersburg, MD). A 96-well plate was quoted with 50 µL of reduced growth factor basement membrane extract (RGF-BME). RGF-BME was allowed to solidify by incubating at 37°C for 1 h. hRMVECs were plated slowly on the membrane at a density of 3×10^4^ cells/well. The cells were treated with different treatment conditions. Cells in VEGF containing ENDO-growth media served as the positive control. The two negative controls used in this experiment were cells incubated in ENDO-basal media and ENDO-growth media with 100 µM sulforaphane. In order to evaluate the effect of honokiol on the tube formation, different concentrations of honokiol (2.5–40 µM) were added in the ENDO-growth media. Cells were incubated for 18 h at 37°C in a CO_2_ incubator. The media was carefully aspirated and cells were washed with 150 µL PBS. Further to each well 2 µM Calcein AM (in PBS) was added and incubated for 30 min at 37°C. After the incubation Calcein AM was removed and PBS was added. Extent of tube formation was assayed using fluorescence images. Efficacy of honokiol on tube formation was evaluated by quantitating the average branch length using angiogenesis analyzer plugin for ImageJ.

## Results

### Honokiol inhibits HIF-dependent luciferase expression

**Figure 1:**
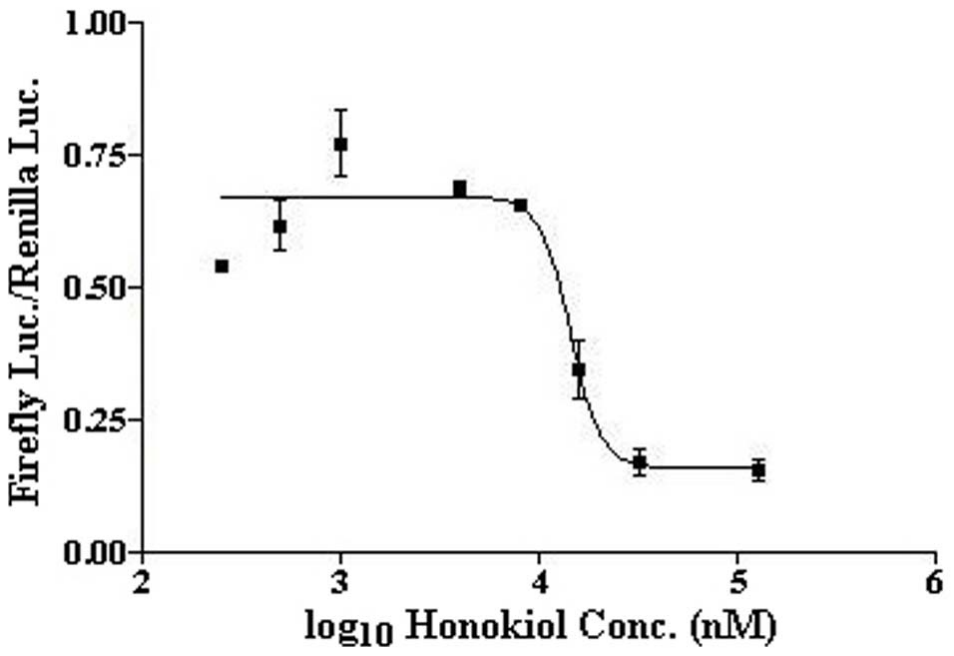
Determination of anti-HIF IC_50_ value of honokiol. HeLa cells were co-transfected with 5HRE-ODD-luc and pSVRenilla plasmids using Lipofectamine 2000 for 24 h, before treatment with 1 mM CoCl_2_ (hypoxia-mimetic agent) under normoxia and different honokiol concentrations (0.2–100 µM) for 12 h. Luminescence was measured using Dual-Luciferase Reporter Assay System. Plotting the relative luciferase activities vs. log_10_ value of honokiol concentrations yielded the anti-HIF IC_50_ value of 14.7 µM for honokiol.

To determine the anti-HIF IC_50_ value of honokiol, we developed a HIF reporter assay. In this assay, due to limited transfection efficiency of RPE cells, HeLa cells were transiently co-transfected (transfection efficiency optimized to ≈80% using Lipofectamine 2000) with 5HRE-ODD-luc and pSVRenilla plasmids. In the reporter plasmid (5HRE-ODD-luc), 5 copies of the HRE (5HRE) and an oxygen-dependent degradation (ODD) domain of HIF-1α regulate the expression of luciferase (luc). Because the ODD domain causes the oxygen-dependent degradation of the ODD-luc protein, this reporter plasmid shows little leakiness of luminescence under normoxia [31]. The HRE contains essential binding sites for HIF that mediates increased transcription in cells that are exposed to hypoxia (or hypoxia-mimetic agents, *e.g.* CoCl_2_ under normoxia). In pSVRenilla plasmid, the SV40 promoter drives the expression of renilla luciferase coding sequences. Thereby its expression is not oxygen dependent. Hence, by determining the ratio of firefly/renilla luciferase activity in HeLa cells exposed to normoxia or normoxia in presence of 1 mM CoCl_2_ (*i.e.* hypoxic conditions), we determined hypoxia-induced firefly luciferase activity and its inhibition by honokiol. These experiments demonstrated that honokiol has an anti-HIF IC_50_ value of 14.7 µM (Fig. 1). This result is consistent with our previous studies using qPCR experiments that honokiol progressively inhibits the HIF pathway in a number of cancer and RPE cell lines at 5–20 µM concentrations [18].

### Mechanism of HIF inhibition by honokiol

**Figure 2:**
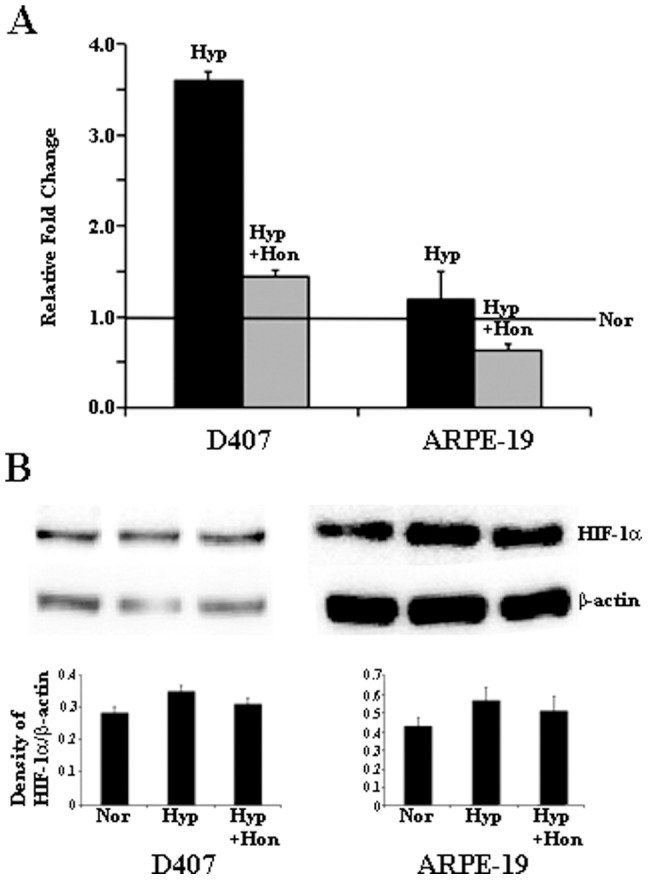
Relative fold change in the mRNA levels of HIF-1α in D407 and ARPE-19 cell lines exposed to hypoxia in presence or absence of honokiol (A). Solid black and grey bars represent relative mRNA fold change ± standard error with or without honokiol, respectively. Normoxic levels are represented by the horizontal line. Western blot analysis of HIF-1*α* protein expression in D407 and ARPE19 cells (B). Lane 1, 2, and 3 represents normoxic sample/Nor, hypoxic sample/Hyp, and hypoxic samples with honokiol/Hyp+Hon. Densitometry was performed with background subtraction and protein bands were normalized against the β-actin band of the same sample.

In order to determine the mechanism of HIF inhibition by honokiol, we evaluated its effect on HIF-1α transcription, translation, translocation to the nucleus, co-localization with HIF-β, and binding of HIF to HRE in RPE cells. To evaluate the effect of honokiol on HIF-1*α* mRNA and protein expression, we performed qPCR and western blot analysis, respectively. We observed very little change in the level of HIF-1α mRNA in ARPE19 cells exposed to normoxia, hypoxia or hypoxia with honokiol (Fig. 2A). Although, these effects were more pronounced in D407 cells. However, when we assayed for the HIF-1*α* protein levels using western blot analysis, only small changes in the HIF-1α protein levels in both RPE cell lines under normoxia, hypoxia or hypoxia treated with honokiol were detected (Fig. 2B). These results were confirmed by performing western blots using two different antibodies (ab1 and ab2185, abcam) for HIF-1*α* (data not shown). Our results of insignificant changes in the HIF-1α protein levels in RPE cells treated with normoxia or hypoxia is consistent with previous published results by *Forooghian et al* in ARPE-19 cells [32]. These results suggest that honokiol does not significantly alter the levels of HIF-1α protein in RPE cells.

**Figure 3:**
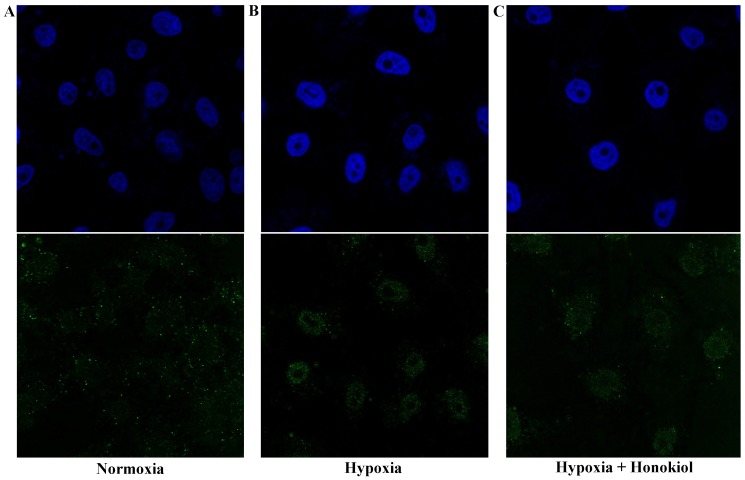
Immunofluorescence analysis to evaluate translocation of HIF-1*α* from cytoplasm to nucleus. D407 cells were stained with anti HIF-1*α* (green), and DAPI (blue). Panel A, B, and C represents normoxic sample, hypoxic sample, and hypoxic samples with 20 µM honokiol.

**Figure 4:**
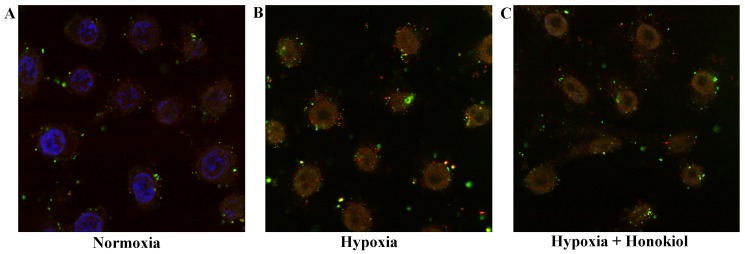
Immunofluorescence analysis to evaluate co-localization of HIF-1*α* and HIF-β in D407 cells. Panel A, B, and C represents normoxic sample, hypoxic sample, and hypoxic samples with 20 µM honokiol. D407 cells were stained with anti-HIF-1*α* (green), anti-HIF-β (red), and DAPI (blue).

Next, we evaluated the translocation of HIF-1α protein into the nucleus and the effect of honokiol on this step. We observed a time-dependent increase in the nuclear accumulation of HIF-1α (highest at 12 h, data for other time points are not shown) under hypoxia in D407 cells. Translocation coefficient (M2) for HIF-1α under hypoxia at 12 h was 0.81, which was statistically significant compared to the normoxic M2 value of 0.63. However, at 12 h we found no significant change in the translocation of HIF-1α upon treatment with honokiol (Fig. 3, M2 value was 0.80). This suggested that honokiol had no effect on hypoxia mediated translocation of HIF-1α into the nucleus. Upon translocation to the nucleus, HIF-1α dimerizes with HIF-β to form an active HIF complex. Therefore, we determine the co-localization of HIF-1α with HIF-β in D407 cells under 12 h of normoxia, hypoxia, and hypoxia with honokiol treatment (Fig. 4). Under normoxia we observed an insignificant co-localization of HIF-1α with HIF-β (co-localization coefficient = 0.69). However, in cells exposed to hypoxia and hypoxia with honokiol we observed a statistically significant co-localization of HIF-1α and HIF-β proteins (co-localization coefficients were 0.82 and 0.81, respectively). This suggests that honokiol does not alter the co-localization of HIF-1α and HIF-β proteins under hypoxic conditions. Taken together, our results in RPE cells suggest that HIF pathway is predominantly regulated by (i) the translocation of HIF-1α protein from cytoplasm to nucleus under hypoxia (Fig. 3) and (ii) its dimerization with HIF-β (Fig. 4), and not by the stability of HIF-1α protein (Fig. 2).

**Figure 5:**
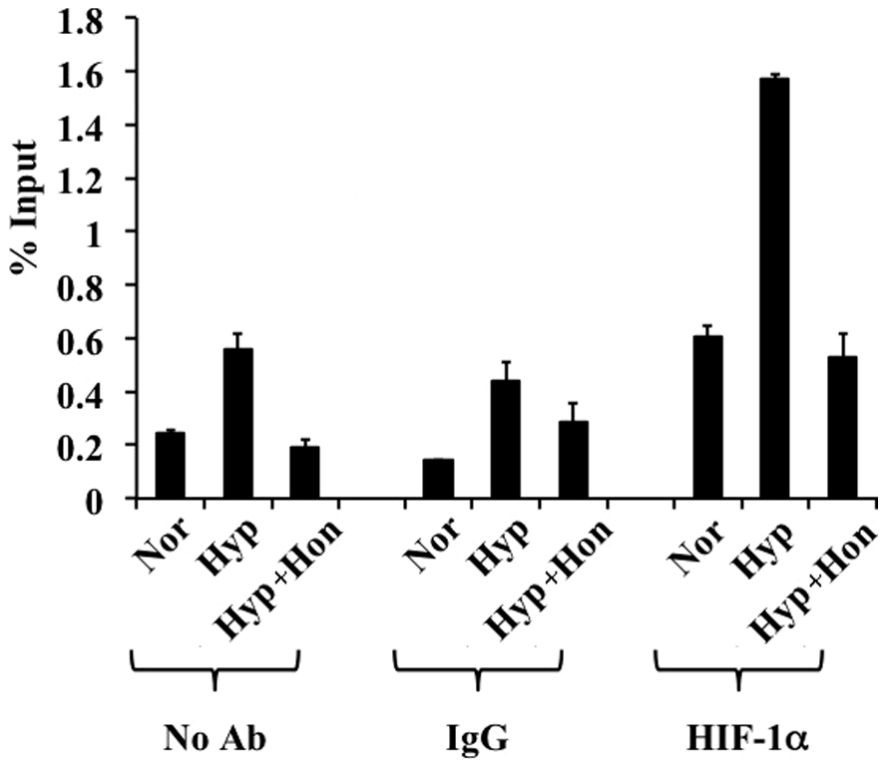
ChIP experiments were performed by exposing D407 cells to (i) normoxia/Nor, (ii) hypoxia/Hyp, or (iii) hypoxia treated with 20 µM honokiol/Hyp+Hon for 16 h. The soluble chromatin was immunoprecipitated using no antibody, non-immune IgG antibody and HIF-1α antibody. Immunoprecipitated DNA was analyzed by qPCR using human VEGF promoter specific primers. Enriched DNA is represented as percentage of the input. Each error bar represents the standard deviation calculated from six experimental replicates.

Activated HIF dimer is recruited to HREs present on VEGF promoter for its transcription under hypoxia. Hence using ChIP, we evaluated the effect of honokiol on HIF-1α occupancy on VEGF HRE under different treatment conditions. These ChIP experiments demonstrated that under hypoxia HIF-1α binding on the VEGF HRE was increased 4-fold compared to normoxia (*i.e.* % input of HIF-1α was 0.3% under normoxia and 1.2% under hypoxia) (Fig. 5). However, when D407 cells were treated with 20 µM honokiol under hypoxia, we observed a very significant (5-fold) reduction in HIF-1α binding to the VEGF HRE (*i.e.* % input of HIF-1α was 0.24). Our negative control experiments without any antibody or non-immune IgG antibody didn't show HIF-1α binding to the VEGF HRE. These experiments demonstrate that honokiol exhibits its anti-HIF activity by inhibiting the binding of HIF-1α to HREs of hypoxia response genes such as VEGF.

### Honokiol inhibits hypoxia mediated VEGF secretion by D407 cells

**Figure 6:**
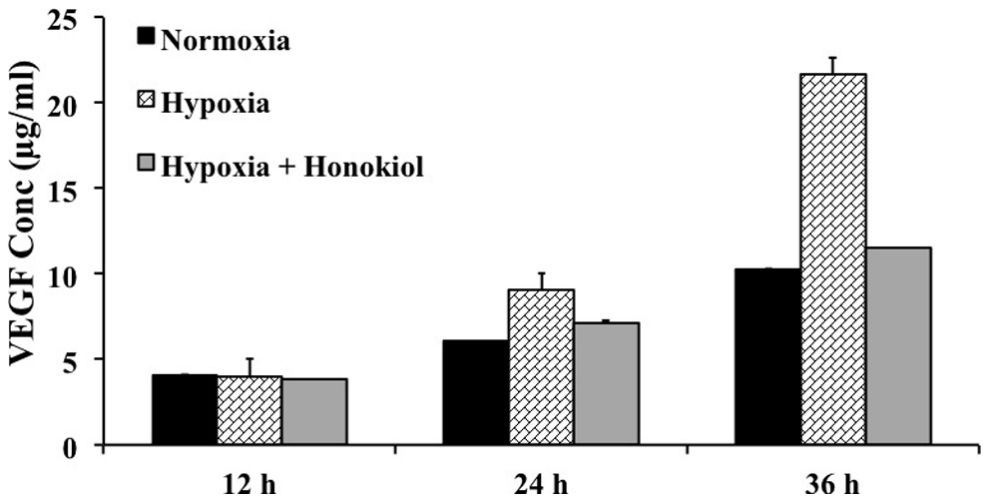
ELISA showing time-dependent secretion of VEGF from D407 cells exposed to normoxia, hypoxia, or hypoxia treated with 20 µM honokiol. Each error bar represents the standard deviation calculated from biological and experimental duplicates.

HIF mediated overexpression of VEGF plays key roles in retinal NV [33]. Therefore, we evaluated the efficacy of honokiol in inhibiting hypoxia mediated VEGF secretion from D407 cells using enzyme-linked immunosorbent assay (ELISA). These experiments demonstrated a time-dependent increase of hypoxia induced VEGF secretion by D407 cells into the cell culture media, peaking at 36 h (25 µg/mL) (Fig. 6). However, treatment of D407 cells with 20 µM honokiol inhibited the VEGF secretion by 50% (12.5 µg/mL) at the same time point (Fig. 6). This confirms that honokiol inhibits VEGF secretion by RPE cells *via* inhibiting the HIF pathway.

### Honokiol inhibits endothelial cell proliferation

Molecular pathogenesis during retinal NV is due to interaction between pro-angiogenic factors (secreted by the activated HIF pathway in astrocytes, RPE, and müller cells) and their receptors present on the retinal micro vascular endothelial cells (RMVECs). This causes an activation of RMVECs and angiogenesis [34], [35], [36]. During the process of angiogenesis, extracellular matrix membrane disintegrates and endothelial cells differentiate into tip and stalk cells. Tip cells proliferate to form filopodia-like protrusive structures, which migrate towards VEGF gradient to form rudimentary vascular tubes. These tubes mature into leaky blood vessels causing retinal NV.

**Figure 7:**
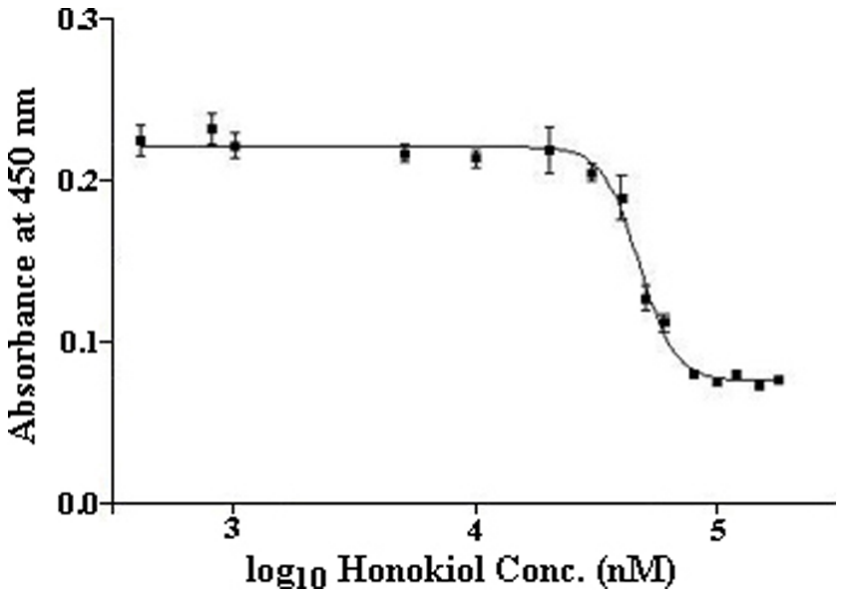
Determination of anti-proliferation IC_50_ value of honokiol. hRMVECs were treated with different concentrations of honokiol (0.4–180 µM of honokiol) for 24 h. The number of viable cells was calorimetrically calculated using the Premixed WST-1 Cell Proliferation Reagent by detecting the amount of formazan formed at 450 nm. Plotting the absorbance at 450 nm vs. log_10_ value of honokiol concentrations yielded the anti-proliferation IC_50_ value of 57 µM for honokiol.

Therefore, direct or indirect anti-angiogenic effects of honokiol on the key steps in retinal NV (*i.e.* endothelial cell proliferation, migration, and tube formation) were evaluated using primary hRMVECs. Initially, the toxicity of honokiol on hRMVEC was determined by culturing these cells in presence of varying concentrations (0.4–180 µM) of honokiol. Based on this we determined the anti-proliferation IC_50_ value of honokiol on primary hRMVEC to be 57 µM (Fig. 7). Hence, we performed all of our *in vitro* anti-angiogenic assays with hRMVECs at concentrations below the anti-proliferation IC_50_ value of honokiol.

**Figure 8:**
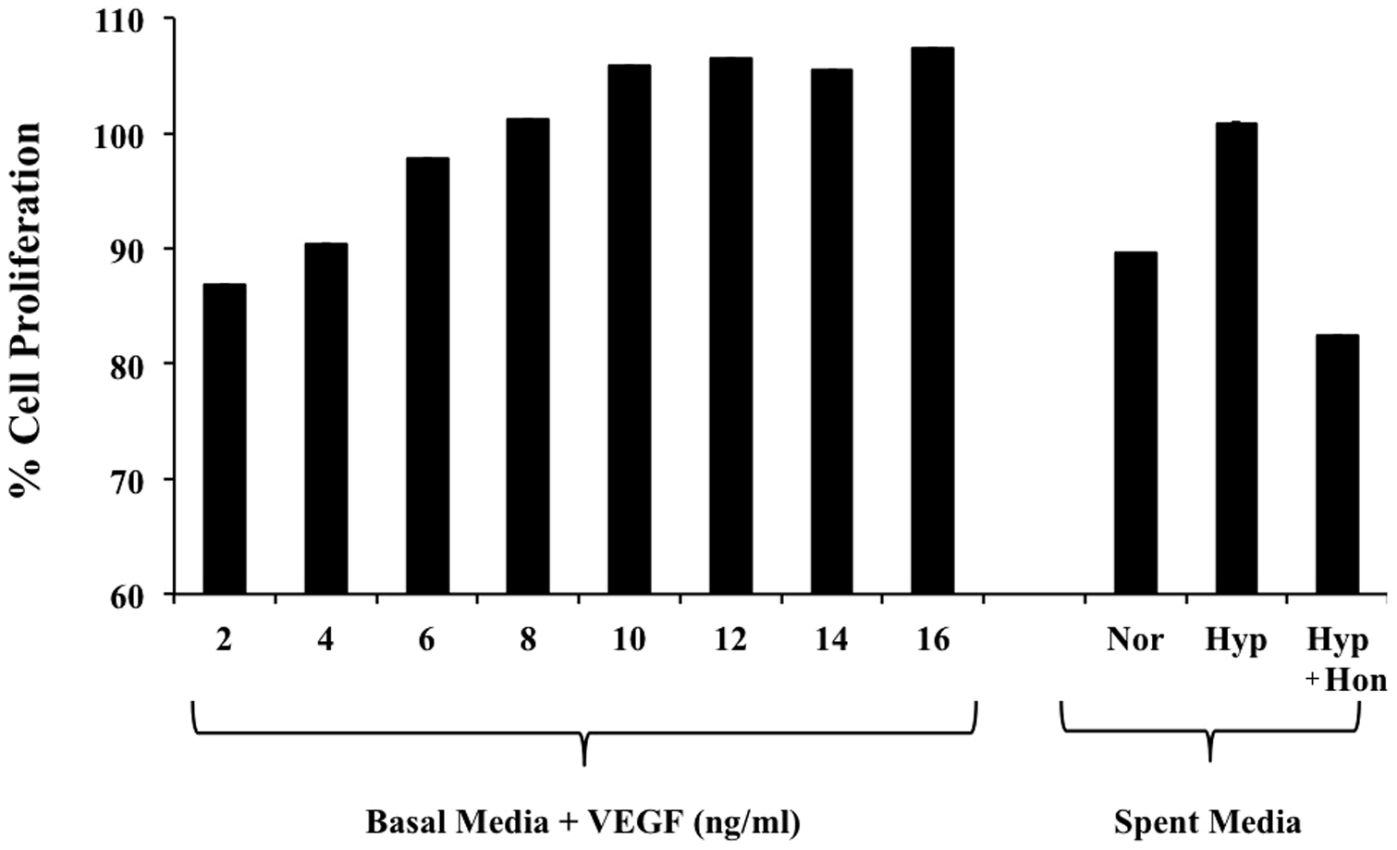
Proliferation of hRMVECs in the presence of 2–16 ng/ml of VEGF (left bars). Right 3 bars represent proliferation of hRMVECs treated with 1000-fold dilutions of spent media from D407 cells exposed to (i) normoxia/Nor, (ii) hypoxia/Hyp, and (iii) hypoxia treated with 20 µM honokiol/Hyp+Hon. Each error bar represents the standard deviation calculated from six experimental replicates.

First, we evaluated the effect of varying VEGF levels in the media (2–16 ng/mL) on proliferation of hRMVECs. These experiments showed a concentration dependent increase in the proliferation of hRMVECs, which plateaued at 12 ng/mL of VEGF (Fig. 8). Using ELISA, VEGF levels in the D407 cells spent media grown under normoxia, hypoxia, or hypoxia treated with 20 µM honokiol were quantified to be 6.5, 9.3, and 5.8 µg/mL, respectively. Thousand fold dilutions of spent media from all three treatments were made to achieve VEGF levels in the linear range where hRMVECs showed a concentration dependent increase in the proliferation. These experiments demonstrated that spent media from D407 cells grown under hypoxia had ∼30% more proliferation compared to normoxic media. Importantly, this increase in proliferation of hRMVECs by the spent media from D407 cells grown under hypoxia was inhibited by ∼40% when hRMVECs were exposed to spent media from D407 cells treated with 20 µM honokiol (Fig. 8).

### Honokiol inhibits in vitro endothelial cell migration

**Figure 9:**
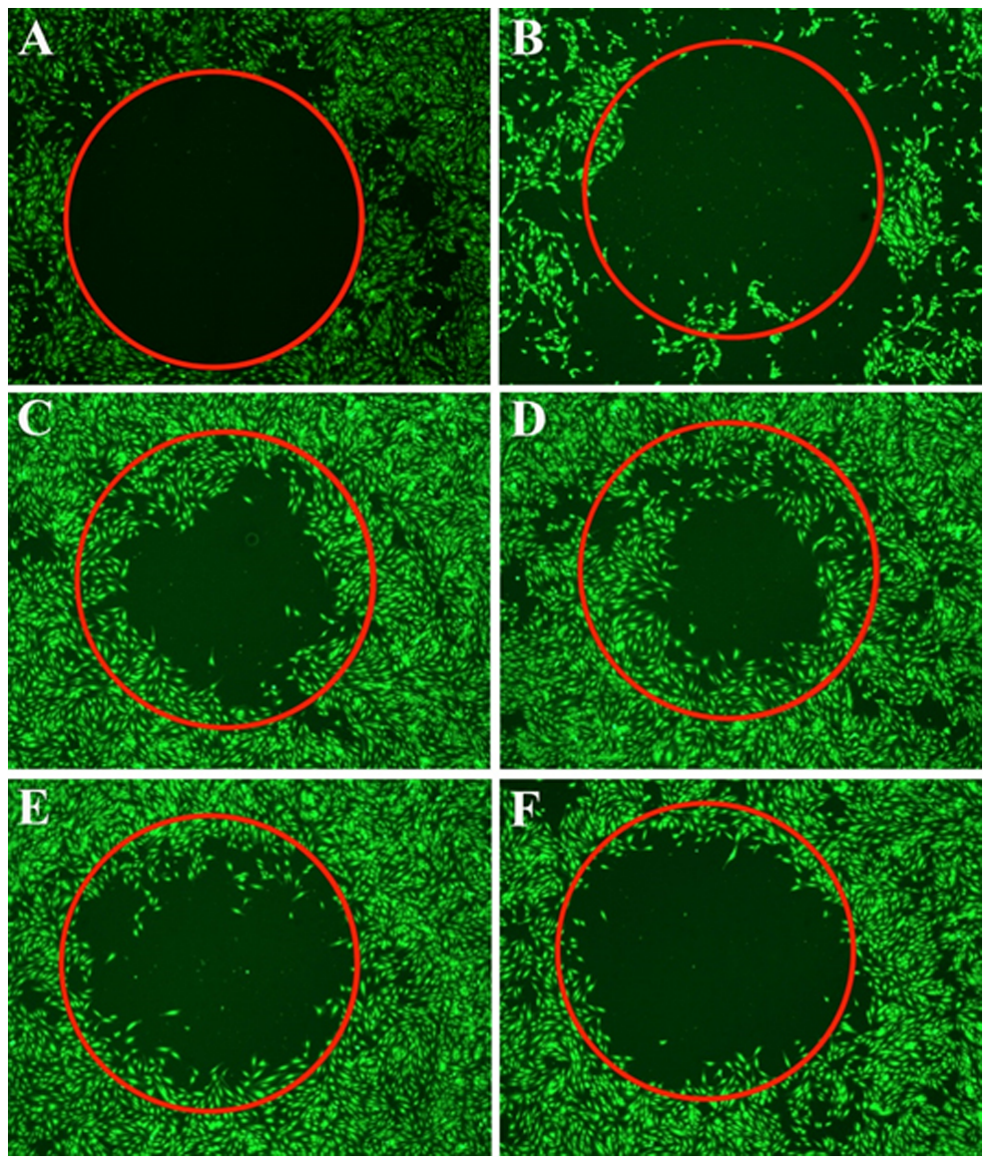
Cell migration assay performed using hRMVECs for 16 h. The cells were treated with the following conditions: (A) Complete media supplemented with 50 ng/ml VEGF at time zero, (B) Basal media,(C) Complete media, (D) Complete media supplemented with VEGF (50 ng/ml), (E) D plus 10 µM honokiol, and (F) D plus 20 µM honokiol.

Initially, we performed hRMVEC migration assay using the collagen-I coated plates for the control samples, *i.e.* (1) basal media (devoid of pro-angiogenic factors including VEGF), (2) complete media (containing pro-angiogenic factors like VEGF), and (3) complete media supplemented with VEGF (50 ng/ml). These experiments showed a VEGF-dependent migration of hRMVECs (Fig. 9A–D), re-establishing the chemo-attractant role of VEGF. To assess the direct effect of honokiol on migration, different honokiol concentrations were added to hRMVECs in the complete media supplemented with VEGF (50 ng/ml). These experiments clearly demonstrated an anti-migratory effect of honokiol at or over 10 µM concentration (Fig. 9E–F). No effect on cell migration was observed at honokiol concentrations below 10 µM (data not shown).

### Honokiol inhibits in vitro endothelial tube formation

**Figure 10:**
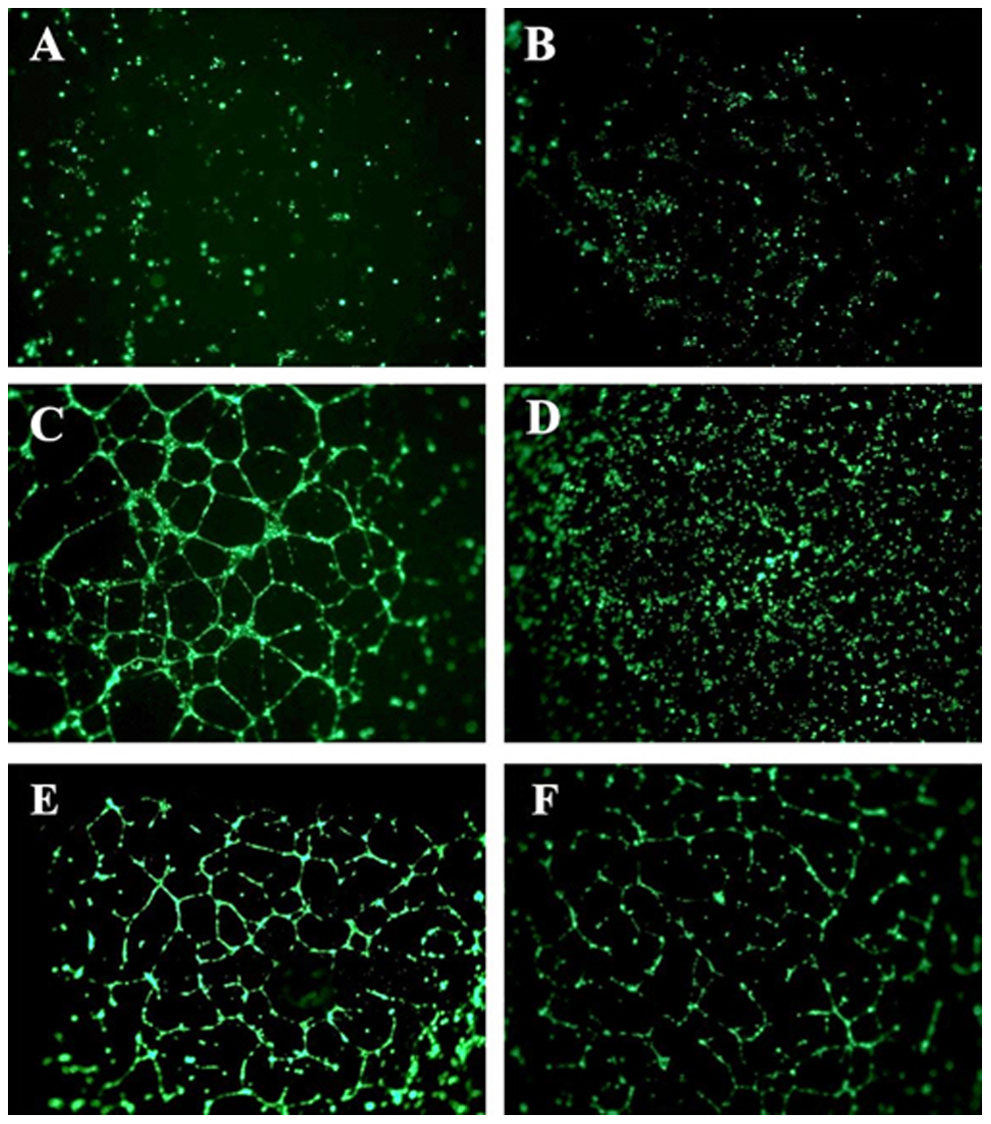
Tube formation assay performed using hRMVECs for 18 h. The cells were treated with the following conditions: (A) ENDO-basal media, (B) ENDO-basal media with VEGF, (20 ng/mL), (C) ENDO-growth media, (D) ENDO-growth media containing 100 µM of sulforaphane, (E) C plus 30 µM honokiol, and (F) C plus 40 µM honokiol.

To evaluate the inhibitory effect of honokiol on the tube forming ability of hRMVECs, we initially performed *in vitro* angiogenesis assay on 3-D Culture Matrix RGF BME (*aka* Matrigel) for control samples, *i.e.* (A) ENDO-basal media, (B) ENDO-basal media with VEGF (20 ng/mL), (C) ENDO-growth media, which contains a complex mixture of growth factors, and (D) ENDO-growth media containing 100 µM of sulforaphane, a known angiogenic inhibitor (Fig. 10A–D). These experiments demonstrated formation of well-networked endothelial tubes only in the ENDO-growth media. This indicates that endothelial tube formation process requires other growth factors along with VEGF. Formation of these rudimentary vascular tubes was inhibited by sulforaphane. No noticeable effect on tube formation was observed at honokiol concentrations below 30 µM (data not shown). When 30 and 40 µM of honokiol was added to the ENDO-growth media, it inhibited the formation of endothelial tubes (Fig. 10E–F). The inhibitory effect of honokiol on tube formation was also observed on the average tube length. In complete media the average tube length was 240, while in cells treated with 30 and 40 µM honokiol it reduced to 160 (data not shown).

## Discussion

Pathological NV is observed in many retinal ischemic diseases (e.g. DR, ROP, RVO, etc.), which is the most prevalent cause of vision loss in working-age Americans. These diseases develop because different primary insults damage retinal blood vessels generating poorly perfused areas. In case of DR, the primary insult is hyperglycemia [37]; in ROP, it is exposure to levels of oxygen that are higher than normal for a particular stage of retinal vascular development [38]; and in RVO, it is obstruction of vascular flow which increases hydrostatic pressure and reduces perfusion [39]. Irrespective of the different underlying reasons, they all ultimately lead to retinal ischemia causing hypoxia, which activates the HIF pathway. Activation of HIF pathway leads to expression of pro-angiogenic factors like VEGF, IGF-1, FGF, SDF-1, PlGF, PDGF, etc. [40]. In the eye these pro-angiogenic growth factors (secreted by RPE cells, astrocytes, and müller glial cells) bind to the receptors on RMVECs, leading to the activation of the endothelial cells [34], [35], [36]. This activation is followed by disintegration of extracellular matrix membrane and differentiation of endothelial cells into tip and stalk cells. Tip cells proliferate and form filopodic structures, migrating towards VEGF gradient that end up forming into tube like structures. These structures mature into rudimentary blood vessels. During the initial stages of retinal NV the blood vessels are not leaky and they grow on the retinal surface. This condition is termed as intra-retinal micro vascular abnormality [40]. In the next step, the blood vessels grow aberrantly and spread towards the outer surface reaching vitreous layer; connecting vitreous layer to the retinal surface. This developed network lacks tight junctions and leak plasma leading to vitreous degeneration, shrinkage and contraction. Contraction of vitreous pulls the retinal surface along with it, causing retinal damage and detachment resulting in blindness [40].

HIF pathway also plays a critical role in the angiogenesis and metastasis of solid tumors [41]. Recent studies have demonstrated a critical role of HIF-1α in the maintenance of cancer stem cells [42], [43]. HIF inhibitors can have a significant therapeutic impact on the treatment of retinal angiogenesis and cancers. As a result, a growing number of chemical compounds are identified that inhibit HIF activity through a variety of molecular mechanisms, including inhibition of HIF-1α transcription (*e.g.* aminoflavone [44]), inhibition of HIF-1α translation (*e.g.* rapamycin [45], digoxin [46], 2-methoxyestradiol [47], topotecan [48], NSC-644221 [49], EZN-2968 [50], etc.), increased HIF-1α degradation (*e.g.* 17-allylamino-17-demethoxygeldanamycin [51], N-acetyl cysteine [52], PX-12 [53], LAQ824 [54], berberine [55], Se-methylselenocysteine [56], YC-1 [57], etc.), decreased HIF subunit heterodimerization (*e.g.* acriflavine [58]), decreased HIF binding to DNA (*e.g.* doxorubicin, daunorubicin [59], echinomycin [42], etc.), and decreased HIF transcriptional activity (*e.g.* bortezomib [60]). A number of these compounds are either in clinical trials or are already approved for the treatment of cancer or other diseases.

We have earlier shown that pure honokiol inhibits the HIF pathway and hypoxia-mediated expression of pro-angiogenic genes in a number of cancer and retinal pigment epithelial (RPE) cell lines [18]. Since pathological activation of the HIF pathway leading to expression of pro-angiogenic genes is the fundamental cause of ocular neovascularization (NV) [40], we have used an OIR model to evaluate the anti-HIF and anti-angiogenic properties of honokiol [23]. This model has been extensively used to study the regulation of angiogenic factors, vascular pathogenesis, and the efficacy of anti-angiogenic compounds [24], [61]. In this model, the P7 mouse pups are placed in 75% oxygen for 5 days. Exposure of 7 day old pups to high levels of oxygen inhibits retinal vessel growth and causes significant oxygen-induced central retinal vessel loss (*i.e.* vaso-obliteration). At P12, the mice are returned to room air and the non-perfused central retina becomes relatively hypoxic. This activates the HIF pathway and pathological neovascularization, which is maximal at P17 [62]. We have shown that daily intraperitoneal injection of honokiol starting at P12, a time point when the HIF pathway and pathological neovascularization gets activated, in an OIR mouse model significantly reduced retinal neovascularization at P17 [23]. Administration of honokiol also prevents the oxygen-induced central retinal vaso-obliteration, characteristic feature of the OIR model. Honokiol also enhanced physiological revascularization of the retinal vascular plexuses.

In the current study, we have evaluated the mechanism of HIF inhibition by honokiol in RPE cells and its anti-angiogenic properties on hRMVECs because, as stated above, in the eye pro-angiogenic growth factors secreted by RPE and other cells bind to the receptors on RMVECs, leading to their activation causing pathological retinal neovascularization. We demonstrated that honokiol inhibits binding of HIF to hypoxia-response elements present on VEGF promoter. We further showed that honokiol inhibits secretion of VEGF protein by RPE cells under hypoxic conditions in a time-dependent fashion. This lower secreted VEGF levels resulted in reduced proliferation of hRMVECs. Then using endothelial cell migration and tube formation assays we showed that, in addition to anti-HIF effect, honokiol manifests potent anti-angiogenic effect on hRMVECs. Thus, our results demonstrate that honokiol possesses both anti-HIF and anti-angiogenic properties. Honokiol has a better efficacy profile as a HIF inhibitor compared to digoxin and doxorubicin [23], the other two HIF inhibitors from natural sources that are evaluated for ocular neovascular diseases [24], [25]. The crude extracts, containing honokiol, from the bark and/or seed cones of *Magnolia* plants have been extensively used for thousands of years in the traditional oriental medicine [19]. In addition, honokiol is a neuroprotective agent with no known toxicity [20], [21]. Therefore, clinical trials should begin to test the hypothesis that incorporation of honokiol into current regimen will benefit patients suffering from cancers and ocular retinopathies.
